# Supplementary material for: Effect of a Practice-Oriented Electronic Medical Record Education Program for New Nurses
Source: Healthcare (Basel). 2025 Feb 8;13(4):365. doi: 10.3390/healthcare13040365 (PMC11855219; doi:10.3390/healthcare13040365)
Supplement: Supplementary file 1 [file healthcare-13-00365-s001.zip › healthcare-3432614-supplementary.pdf]

**Section 1: Computerized Nursing Competency for Inpatient Admission**

**▣ Please mark the appropriate response (✓) for each question.**

| No. | Item | Not at all proficient | Not proficient | Neutral | Proficient | Highly proficient |
|---|---|---|---|---|---|---|
| 1 | I can accurately document the actual admission time of a patient. | | | | | |
| 2 | I can update the current location of a patient upon admission. | | | | | |
| 3 | I can verify the patient's personal information, including phone number. | | | | | |
| 4 | I can print identification labels such as name tags and wristbands. | | | | | |
| 5 | I can correctly enter dietary prescriptions according to medical orders. | | | | | |
| 6 | I can input necessary information into the clinical observation record. | | | | | |
| 7 | I can complete the initial nursing assessment without omissions. | | | | | |
| 8 | I can review and verify various consent forms. | | | | | |
| 9 | I can perform medication identification requests according to protocol. | | | | | |
| 10 | I can conduct patient safety evaluations, including fall and pressure ulcer risk assessments. | | | | | |
| 11 | I can report pressure ulcers when present. | | | | | |
| 12 | I can perform an initial pain assessment. | | | | | |
| 13 | I can record nursing activities appropriately according to the situation. | | | | | |
| 14 | I can utilize nursing handover reports according to patient status and treatment plans. | | | | | |
| 15 | I can write nursing records using nursing assessment, goals, interventions, and evaluation. | | | | | |
| 16 | I can verify prescribed medical orders and accurately perform action checks. | | | | | |
| 17 | I can prescribe necessary treatment materials for patients. | | | | | |

**Section 2: Computerized Nursing Competency for Surgical and Procedural Nursing Care**

**▣ Please mark the appropriate response (✓) for each question.**

| No. | Item | Not at all proficient | Not proficient | Neutral | Proficient | Highly proficient |
|---|---|---|---|---|---|---|
| 1 | I can accurately verify surgical and procedural consent forms. | | | | | |
| 2 | I can retrieve surgery and procedure schedules. | | | | | |
| 3 | I can confirm the procedure name in the pre/post-surgical nursing record menu. | | | | | |
| 4 | I can correctly enter vital signs in the preoperative nursing record. | | | | | |
| 5 | I can accurately input preoperative preparation details. | | | | | |
| 6 | I can record the surgical room transfer time in the nursing log. | | | | | |
| 7 | I can document the medical equipment and supplies sent to the operating room. | | | | | |
| 8 | I can input postoperative nursing care information correctly in the surgical care menu. | | | | | |
| 9 | I can select the appropriate procedure name in the procedural safety record. | | | | | |
| 10 | I can accurately document information in the procedural safety record. | | | | | |
| 11 | I can match the recorded procedure time with the nursing log. | | | | | |
| 12 | I can complete the handover documentation for patient transfer to the operating room. | | | | | |
| 13 | I can verify and execute pre- and post-procedural medical orders. | | | | | |
| 14 | I can correctly document required pre- and post-procedural information in the nursing log. | | | | | |
| 15 | I can manage drainage tubes and wound dressings accurately. | | | | | |
| 16 | I can prescribe necessary treatment materials for patients undergoing surgery or procedures. | | | | | |
| 17 | I can accurately reassess patients for falls and pain post-procedure. | | | | | |

**Section 3: Computerized Nursing Competency for Patient Transfer, Relocation, and Discharge**

▣ **Please mark the appropriate response (✓) for each question.**

| No. | Item | Not at all proficient | Not proficient | Neutral | Proficient | Highly proficient |
|---|---|---|---|---|---|---|
| 1 | I can request a change of department for a patient. | | | | | |
| 2 | I can document patient transfers in the nursing log. | | | | | |
| 3 | I can update administrative location changes. | | | | | |
| 4 | I can complete transfer records without omissions. | | | | | |
| 5 | I can update the patient's current location after admission. | | | | | |
| 6 | I can verify and acknowledge transfer records upon patient admission. | | | | | |
| 7 | I can print patient identification labels and wristbands. | | | | | |
| 8 | I can perform location updates and transfer documentation for intra-ward movements. | | | | | |
| 9 | I can apply isolation room status and correctly enter the isolation reason and level. | | | | | |
| 10 | I can verify meal discontinuation and adjust meal orders accordingly. | | | | | |
| 11 | I can check outpatient appointment availability and schedule follow-ups. | | | | | |
| 12 | I can verify outpatient appointment dates and adjust discharge medication days accordingly. | | | | | |
| 13 | I can modify scheduled test appointment dates in the discharge orders. | | | | | |
| 14 | I can document discharge-related records in the nursing log. | | | | | |
| 15 | I can complete and print the discharge nursing records. | | | | | |
| 16 | I can check and confirm the discharge progress. | | | | | |
| 17 | I can retrieve and print medical certificates, insurance documents, and physician notes. | | | | | |

**Section 4: Computerized Nursing Competency for Night Duty Tasks**

▣ **Please mark the appropriate response (✓) for each question.**

| No. | Item | Not at all proficient | Not proficient | Neutral | Proficient | Highly proficient |
|---|---|---|---|---|---|---|
| 1 | I can document nursing notes based on patient conditions after ward rounds. | | | | | |
| 2 | I can perform pain assessments. | | | | | |
| 3 | I can compare previous and current medical orders for task verification. | | | | | |
| 4 | I can print tickets for injections and nebulizers according to schedule. | | | | | |
| 5 | I can print insulin administration tickets. | | | | | |
| 6 | I can generate barcodes for required blood and glucose tests. | | | | | |
| 7 | I can order necessary medical supplies and materials. | | | | | |
| 8 | I can print patient lists for weight measurements, X-ray exams, dressing procedures, and BST checks. | | | | | |
| 9 | I can review the list of scheduled functional tests (e.g., EKG, PFT, endoscopy, 2D-Echo). | | | | | |
| 10 | I can request sterile equipment and small medical tools. | | | | | |

* BST= Blood sugar test; EKG= Electrocardiography; PFT= Pulmonary function test.

**Section 5: Computerized Nursing Competency for SBAR and Handover**

**▣ Please mark the appropriate response (✓) for each question.**

| No. | Item | Not at all proficient | Not proficient | Neutral | Proficient | Highly proficient |
|---|---|---|---|---|---|---|

| | | | | | | |
|---|---|---|---|---|---|---|
| 1 | I can identify the reason for patient admission. | | | | | |
| 2 | I can review past medical history, including medication history. | | | | | |
| 3 | I can input and assess changes in vital signs. | | | | | |
| 4 | I can check and review test results. | | | | | |
| 5 | I can verify interdepartmental consultations and referral responses. | | | | | |
| 6 | I can confirm diagnosis and treatment plans. | | | | | |
| 7 | I can track previous medical history. | | | | | |
| 8 | I can review outpatient and emergency department records. | | | | | |
| 9 | I can check and document the presence and condition of indwelling medical devices. | | | | | |
| 10 | I can verify physicians' orders and interpret relevant instructions. | | | | | |
| 11 | I can record necessary information for nursing handover. | | | | | |
| 12 | I can utilize SBAR for effective nursing handover. | | | | | |
| 13 | I can use SBAR for effective communication with other healthcare professionals (e.g., physicians). | | | | | |

* SBAR = Situation, Background, Assessment, and Recommendation.
